# Characteristics of Pulsed-Laser-Induced Layers with Cracks Prepared for SiC Grinding Processes

**DOI:** 10.3390/ma19020397

**Published:** 2026-01-19

**Authors:** Hu Li, Yanjiao Jiang, Yujia Yang, Jianyu Yang, Lida Zhu

**Affiliations:** School of Mechanical Engineering and Automation, Northeastern University, Shenyang 110819, China

**Keywords:** SiC, laser modification, grinding, surface hardness, crack

## Abstract

**Highlights:**

**What are the main findings?**

- The dimensions of the laser-induced crack layer can be controlled.
- The dimensions of the controlled cracks contribute to a decrease in grinding force and specific grinding energy.

**What are the implications of the main findings?**

- Controlling the dimensions of the laser-induced layer helps reduce material consumption in silicon–carbon splitting during separation processes.
- The grinding characteristics of the rough surface after silicon–carbon separation improve the quality of the ground surface.
- Pulsed-laser parameters play an important role in material separation.

**Abstract:**

When grinding silicon carbide, surface and subsurface damage have a significant impact on the product’s surface quality. One method to control the crack dimensions is laser irradiation on the SiC surface. The effect of this method on the grinding process is analyzed in this study. A series of experiments was carried out based on an orthogonal experimental design, with systematic adjustments made to laser parameters, including pulse energy (current), laser spot spacing, scanning times, and grinding process parameters. During the experiments, the grinding force was monitored by a dynamometer, and the specific grinding energy was calculated accordingly. Pulsed engraving laser modification effectively reduced the hardness of the ceramic surface layer by about 20%. The median and radial crack sizes induced by the laser in the subsurface layer ranged from 20.4 μm to 54.3 μm. This effectively inhibited further propagation of median and radial cracks during the grinding processes. Simultaneously, the tangential grinding force *F_t_* was reduced by 30%. These conclusions were obtained through corresponding experiments that link surface roughness to laser power and grinding parameters. Using laser-induced controllable crack characteristics in the grinding process allow damage from surface and subsurface grinding to be controlled in brittle materials.

## 1. Introduction

Silicon carbide (SiC) is a hard and brittle material that is challenging to machine. Grinding often induces subsurface damage, high-pressure phase transformations, cracks, brittle fractures, and residual stresses, all of which compromise the integrity of the machined surface. In particular, chipping of the material edges, surface cracks, and pits is detrimental to the performance parameters of machined parts, especially their fatigue life. Reducing surface defects during machining or grinding has been a core purpose of machining research [1,2,3,4]. The brittle material damage model proposed by Lawn and Wilshaw [5] is the primary basis for analyzing subsurface cracks.

Many scholars have been committed to applying grinding technology to SiC surface processing. Kizaki et al. [6] proposed ultraviolet laser-assisted precision cutting of zirconia ceramics, and the results showed that the introduction of a laser significantly reduced the number of macro-cracks and decreased the specific cutting energy by 35%. Azarhoushang et al. [7] successfully applied an ultra-short-pulse laser in the silicon nitride grinding process, achieving efficient material removal, controlling thermal damage, and improving the material removal rate through laser radiation.

The combination of laser and traditional machining methods has given rise to a variety of hybrid machining approaches, including Laser and Ultrasonic Vibration-Assisted Machining (LVC) [8], Laser-Assisted Machining (LAM) [9,10,11,12,13,14,15], laser-assisted drilling [16], laser-assisted grinding [8,15,17,18], and laser cutting [14,19,20]. Among these approaches, scratch tests using single abrasive grains are typical.

The primary objectives of machining are to achieve high surface quality, low power consumption, and reduced tool wear [9,21,22,23,24]. Research on surface quality focuses on inhibiting the propagation of subsurface brittle fracture and expanding the plastic deformation zone [23,25]. Meanwhile, studies on chip morphology and failure modes are an important part of machining mechanism research [8,23]. Based on theories of fracture mechanics [9,23,26], the brittle–ductile transition and critical transition scale are key to surface quality control [9,13,21,27]. Consequently, relevant research on crack initiation, propagation, prefabricated cracks [28], and crack control [13] has been successfully carried out. Further analysis at the crystal level involves aspects such as slip, stacking, stacking faults, and amorphization [13,29,30].

Crack initiation and propagation are typically induced by force/stress [8,9,21,22], which is not easily observed during the machining process. By means of SEM, EDS, and white light interferometry, geometric characteristics can be detected, including surface topography [9,16,18,26], crack distribution, and surface defects [6,7].

Fracture theory is usually based on indentation fracture experiments. Methods adopted in experiments include indentation tests [4], control experiments (conventional cutting) [9,21], Taguchi methods (for analyzing cutting forces and surface quality under different machining conditions) [12], Box–Behnken experimental design [23], response surface methodology (RSM) [23], and design of experiments (DoE) [28]. In these experiments, characterizations are usually based on morphology [9], surface defects, and roughness [8,9,21,27]. At the microscale, detections are carried out on cracks/microcracks [9,21,22,26] and surface/subsurface damage [7,25]. In addition, measurements or calculations are conducted for force/stress [8], specific grinding energy [6,22], hardness [28], etc.

Experiments are conducted under limited conditions, and theoretical analysis helps generalize the experimental results. In this regard, commonly used theoretical analyses include thermo-mechanical coupling simulation [31], temperature field simulation [17], finite element models (FEMs) [8], molecular dynamics simulation [30], surface roughness models [12,23,32], subsurface damage scale models [25], grinding force prediction models [23], and tool wear models [33].

Rao et al. [34] analyzed the mechanism for removing material from grinding surfaces during the grinding process of RB-SiC. They found that grinding surfaces are mainly characterized by brittle pits, abrasive scratches, and plastic grooves. Chen et al. [30] investigated the variation laws of stress, temperature, dislocation, surface topography, and crystal structure in single-crystal and polycrystalline silicon carbide. Changes were observed in grinding speed and grain size during molecular dynamic simulations.

Qu et al. [35] studied material removal from single-crystal 4H-SiC during the grinding process and found that it is mainly achieved through plastic deformation, accompanied by brittle fracture.

To date, extensive research [36,37,38] has been conducted on the grinding process of silicon carbide, including the micro-level grinding removal mechanism and the proposal of various new process methods such as composite grinding. This study investigates strategies to reduce damage and improve processing efficiency during SiC machining by using an engraving laser to modify the material surface of silicon carbide, exploring the mechanism of laser irradiation on the surface properties of silicon carbide. This includes controlling the size of cracks on the surface of laser-modified silicon carbide, restricting the extension and propagation of radial cracks and median cracks, and analyzing the effect that surface cracks on laser-modified silicon carbide have on the grinding process.

## 2. Experimental Section

### 2.1. Sample Surface Preparation

A green laser with a wavelength of 532 nm was used as the internal carving light source (PHANTOM I laser internal carving machine produced by Han’s Laser Group, Shenzhen, China). The silicon carbide ceramic sheets produced by the pressure-less sintering process (Beilong Electronics Co., Ltd., Guangzhou, Guangdong, China) were irradiated. The laser was focused beneath the sample’s surface layer, where thermal cracks modified the targeted region and the performance parameters of the ceramics sample are shown in Table 1.

A pulsed engraving laser processing system was used to irradiate the surface of the silicon carbide ceramics; the specific parameters of the laser engraving machine are listed in Table 2. The laser output power was adjusted by changing the output current; the laser defocus value was changed by adjusting the height of the processing platform in the *Z*-axis direction; and the hardness and microcrack size of the laser-induced layer were altered by adjusting the spot spacing and scanning times of the laser output. The experiment adopted a four-factor and four-level orthogonal experimental method, completing 16 groups of experiments. For each group of experimental conditions, the same experiment was repeated 4 times; the maximum deviation was excluded, and the average value was taken for calculation.

The hardness of the laser-modified layer on silicon carbide ceramic was measured, and the micro-topography was observed. A digital micro-Vickers hardness tester (HVS-1000M, Lead Instrument Co., Ltd., Ningbo, China) was used to measure the microhardness of the laser-irradiated region. A super-depth microscope (VHX-1000, Keyence Corporation, Osaka, Japan) was employed to observe crack topography. The microscope is equipped with low- and high-magnification lenses, with the low-magnification lens capable of magnifying up to 200× and the high-magnification lens magnifying up to 5000×. Additionally, the microscope can perform depth-of-field synthesis on the measured area to automatically generate a 3D surface profile map within the range.

**Figure 1:**
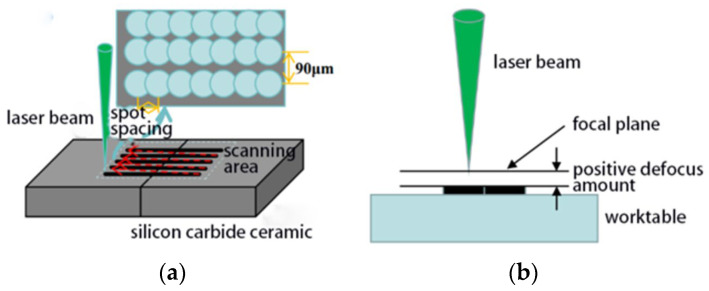
Schematic diagram of laser-modified SiC ceramic. (**a**) Scanning strategy. (**b**) Positive defocus processing.

The phase composition of the laser-modified layer on silicon carbide ceramic was analyzed using an X-ray diffractometer (smartlabX, Lihua Saisi Technology Co., Ltd., Beijing, China), with an angular resolution of 0.001°, and was able to precisely identify the phase composition based on diffraction principles. In the experiment, two silicon carbide ceramic wafers were bonded together with wax to facilitate the observation of cross-section surface and the depth of the thermal modification layer, as shown in Figure 1.

By adjusting the current, the output power of the laser engraving machine can be changed; therefore, the current parameter is used to characterize the laser power. The laser current, number of scans, defocus amount, and spot spacing were selected as the laser process parameters to optimize the control of the microcrack scale in the machined area and its characterized surface hardness.

**Figure 2:**
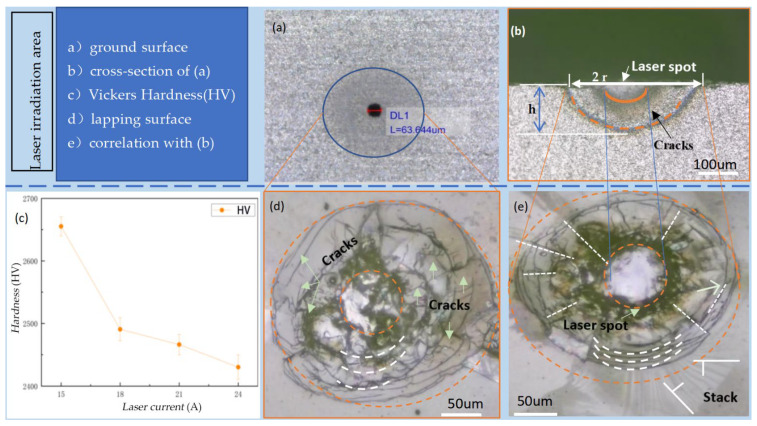
Crack characteristics of SiC ceramic surface layer. (**a**) Ground surface with measured laser spots. (**b**) Cross-section of (**a**). (**c**) Hardness of sample measured by Vickers hardness tester. (**d**) Correlation surface of sample after lapping. (**e**) Another correlation surface of sample after lapping.

Thermal-induced cracks were generated on the material surface using the internal engraving laser, which has a significant impact on the material’s hardness. Material hardness is an important property that affects the grinding process. In additional, the surfaces of the SiC specimens were treated by two processes: grinding and lapping. To enhance the laser energy absorption rate of the specimen surfaces, a grinding treatment was performed using a #400 diamond grinding wheel with *v_sr_* = 10,000 r/min and *v_f_* = 5 mm/min. To observe clear cracks on the specimen surfaces, a lapping treatment was conducted with a #1000 diamond lapping plate, where *v_sr_* = 600 r/min and *v_fr_* = 60 r/min. After laser irradiation, the relevant experimental results are shown in Figure 2a–e.

### 2.2. Grinding Experiment Scheme

**Figure 3:**
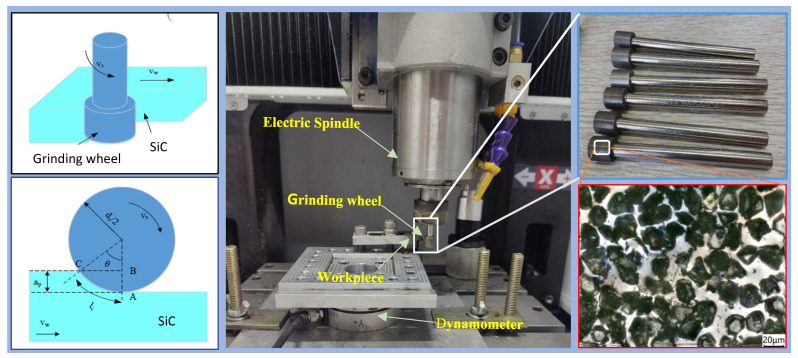
The grinding test bench and abrasive tool.

To verify whether the laser modification method for ceramic surface layers can effectively reduce grinding force and prevent crack propagation, a high-speed precision engraving machine (Mikael 300Q, Mikael CNC Technology Co., Ltd., Shenzhen, Guangdong, China) was used with a #400 diamond electroplated grinding head. The grinding test bench and abrasive tool are shown in Figure 3.

A dynamometer (KWR75, Kunwei, Technology Co., Ltd., Guangzhou, China) was used to measure the grinding force of the process, as shown in Figure 3. Diamond wheel grinding remains the primary grinding method for silicon carbide ceramics. Electroplated grinding wheels offer the advantage of high bonding strength, so an electroplated diamond wheel with an abrasive grain size of #400 and a wheel diameter of 10 mm was selected for this experiment, as detailed in Table 3.

The grinding force generally acts in one of three directions: the normal grinding force *F_n_*, tangential grinding force *F_t_*, and axial grinding force *F_a_*. However, *F_a_* is usually small, and its impact on the machining process can be neglected. In the experiment, the grinding force was measured three times for each set of process parameters. Abrupt signals in the grinding force data were eliminated, and the average value of the grinding force during the stable grinding stage was taken as the final result to avoid the influence of contingency and random errors on the test outcomes.

## 3. Results

### 3.1. Crack Dimensions

**Figure 4:**
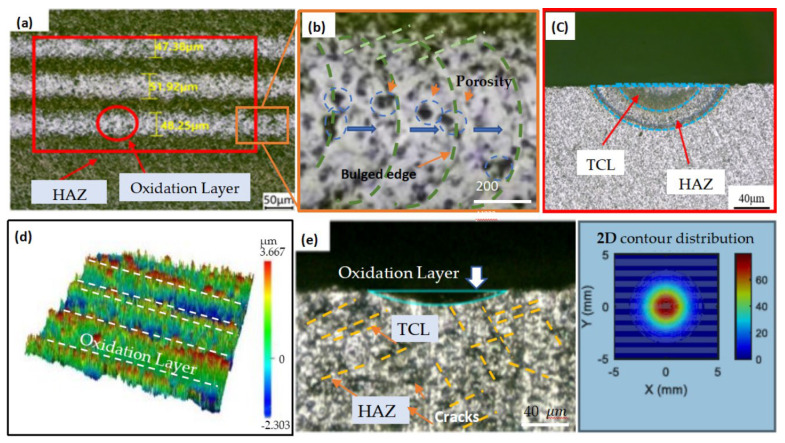
(**a**) Surface topography of laser-modified SiC ceramics. (**b**) Local magnification of (**a**). (**c**) Cross-section of (**a**). (**d**) Three-dimensional topography from super-depth microscope. (**e**) Non-grinding cross-section surface.

The surface topography of SiC ceramics treated by an internal carving laser is shown in Figure 4. The laser-modified layer consists of pulsed laser spots, a thermal crack layer (TCL), and a heat-affected zone (HAZ). The diameter *d* and depth h of the TCL are core indicators of the effect of laser surface treatment, as they reflect the direct influence of the laser on the material’s surface properties (e.g., surface hardness and fracture toughness).

The subsequent grinding process needs to completely remove the TCL and HAZ. Therefore, accurately assessing the thickness of the TCL and the HAZ after laser modification is important.

**Figure 5:**
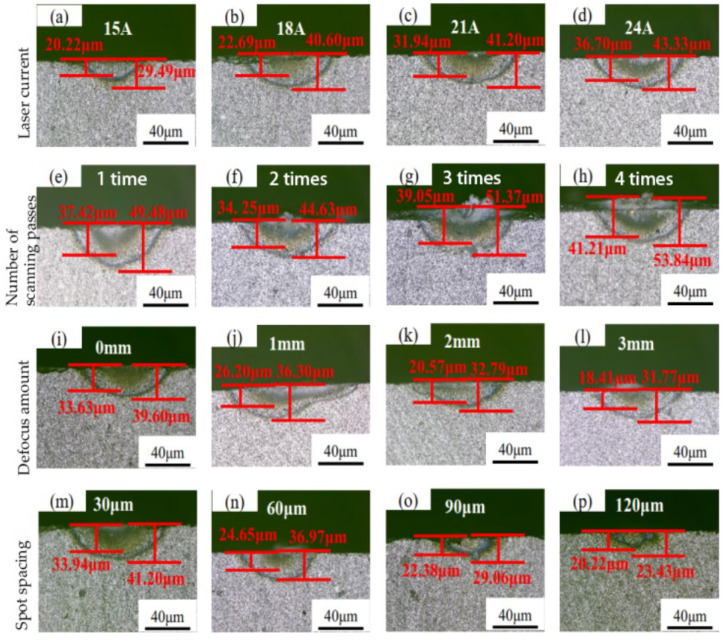
Measuring dimensions of the TCL and HAZ. (**a**–**d**) The dimension of TCL and HAZ under different current. (**e**–**h**) The dimension of TCL and HAZ under different scanning times. (**i**–**l**) The dimension of TCL and HAZ under different defocus amount. (**m**–**p**) The dimension of TCL and HAZ under different spot spacing.

The cross-sectional topography of silicon carbide was measured, which was influenced by laser parameters including laser current, scanning times, defocus value, and spot spacing and was shown in Figure 5. It can be observed that laser parameters have a significant impact on the depths of the TCL and the HAZ. When the laser current is 15 A, the depths of the TCL are close to those of HAZ. As the laser current increases to 18 A, the boundary between the TCL and the HAZ becomes clearer. When the laser current further increases to 21 A and 24 A, due to the increased laser energy density, the surface oxide layer gradually thickens, and a white oxide layer can be clearly observed in the laser-modified zone.

As the number of scans increases from 1 to 4, the depths of the TCL and HAZ gradually increase, while the surface oxide layer also thickens progressively. When the defocus amount increases from 0 mm to 1 mm, the dimensions of the thermal crack zone increase significantly. The reason for this may be that the focal point of positive defocus is located above the sample surface, where the spot diameter formed by the laser beam on the sample surface is larger than that at the focal point. When the defocus amount is further increased to 2 mm and 3 mm, as the distance between the focal point and the sample surface gradually increases, a large amount of energy dissipates in the air, resulting in a decrease in the effective energy transmitted to the material surface, and the width of the modified zone gradually decreases.

When the spot spacing increases from 90 μm to 120 μm, the depths of the TCL are similar to those of HAZ. This can be attributed to the fact that with the increase in spot spacing, the energy absorbed by the material per unit area decreases, leading to reduced thermal accumulation, which, in turn, results in a significant reduction in HAZ comparable to the TCL.

When the number of laser scans reaches four, the silica oxide layer reaches a maximum depth of 20.13 μm, with a TCL depth up to 41.21 μm, and a laser HAZ depth up to 53.84 μm. Spot spacing affects the energy accumulation effect and the continuity of the scanned area. For limited spot spacing, adjacent spots overlap, and the same region is heated by two other pulse spots. Energy accumulation increases the peak temperature, promoting deep thermal diffusion.

### 3.2. Surface Hardness

The Vickers hardness tester was used, with a square pyramid diamond indenter, to measure the hardness of the laser-modified region. After the hardness tester was automatically loaded, held the load, and unloaded, the lengths of the two diagonals for the indentation in the target area were manually calibrated. Subsequently, the hardness tester automatically calculated the corresponding hardness value according to the Vickers hardness empirical formula.

**Figure 6:**
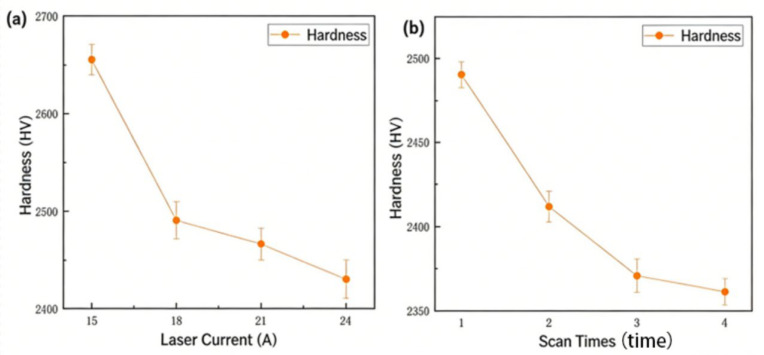
(**a**) The plot of P_Laser_ (indexed by current) on the HV of the laser-modified area. (**b**) Plot of scan times vs. hardness of the laser-modified area.

Increasing the laser current from 15 A to 24 A progressively reduces surface hardness, confirming the strong influence of laser power on material softening, as shown in Figure 6a. As the current increased, the power output by the laser gradually increased, leading to a higher amount of laser energy per unit area. After being transmitted through the optical path and focused onto the silicon carbide surface, part of the laser energy was absorbed by the material, causing the surface temperature to rise.

The surface hardness of silicon carbide gradually decreases as the number of laser scans increases. When the number of scans increases from 1 to 2, the surface hardness of silicon carbide decreases most significantly, and the decreasing trend of surface hardness slows down when the number of scans increases to 3 and 4, as shown in Figure 6b.

### 3.3. Surface Topography and Composition

**Figure 7:**
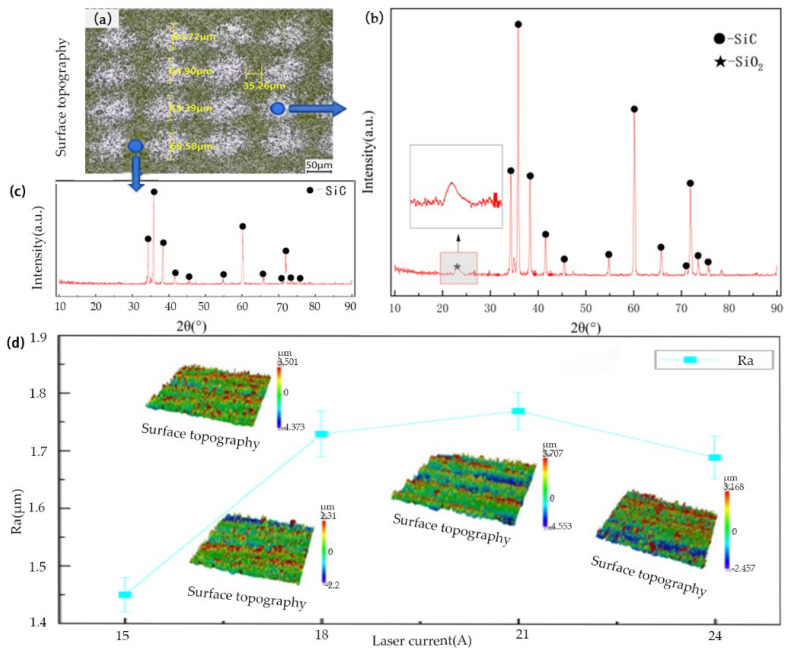
(**a**) Topography of SiC sample; (**b**) XRD spectra of silicon carbide ceramics in the molten pool; (**c**) XRD spectra of silicon carbide out the molten pool; (**d**) the correlation between *R_a_* and laser current.

Since the powdery ablated products generated on the surface during laser irradiation are insufficient for individual detection, XRD analysis was performed on the entire laser-treated surface of the silicon carbide ceramics after laser irradiation. The overall topography of the sample is shown in Figure 7a; the characteristic peaks of the SiC matrix are presented in Figure 7c; and the characteristic peaks of the modified products are illustrated in Figure 7b.

Comparing the XRD spectra of silicon carbide ceramics before and after laser modification, it was found that the characteristic peaks appeared at 2θ = 34.061°, 35.597°, 38.268°, 41.383°, 43.253°, 54.581°, 60.024°, 66.654°, 71.651°, 72.327°, 73.513°, and 76.304°. A comparison with the standard PDF card confirmed that all these characteristic peaks correspond to the SiC phase, indicating that the main constituent phase of the silicon carbide ceramics before laser modification is the SiC phase. After laser modification, in addition to the aforementioned characteristic peaks, a weak-intensity characteristic peak emerges at 2θ = 23.749°, which is identified as the SiO_2_ phase by reference to the standard PDF card. Thus, the main phase generated on the silicon carbide surface after laser modification is the SiO_2_ phase.

Surface roughness measurements of the silicon carbide were conducted, as shown in Figure 7d. The results indicate that when the laser current is 15 A (with low laser power), surface roughness is 1.452 μm. As the laser current increases to 18 A, surface roughness rapidly rises to 1.73 μm, and further increases in the laser current result in minor changes to the surface roughness. At a low laser current, the laser power is insufficient to induce continuous melting of the silicon carbide surface. The roughness decreased in the current of 24 A may be that when the current is further increased, sufficient surface reactions occur, and the surface tension of the molten silicon carbide drives the melt flow to fill the surface microcracks and pores, resulting in the surface roughness ceasing to rise.

### 3.4. Grinding Process

**Figure 8:**
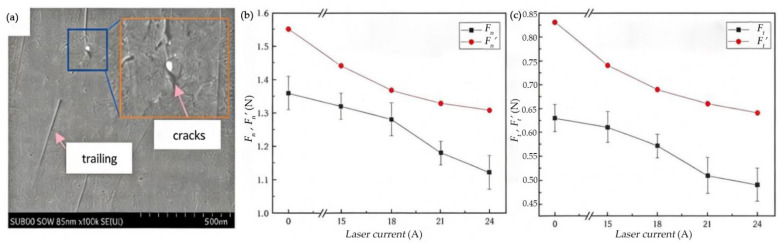
(**a**) SEM topography of the lapping surface. (**b**) The effect of laser power on *F_n_*, $F_{n}^{\prime }$. (**c**) The effect of laser power on *F_t_*, $F_{t}^{\prime }$.

The presence of surface and subsurface cracks significantly reduces the grinding force. As the laser current gradually increases, both the *F_n_*, and *F_t_*, decrease progressively. Compared to the control group $F_{n}^{\prime }$, $F_{t}^{\prime }$ without laser modification, the grinding forces are reduced by varying degrees, as shown in Figure 8. The ground surface undergoes further lapping treatment, and its topography is presented in Figure 8a.

When the laser current is 15 A, the *F_n_* decreases by 2.9% compared with conventional grinding, and the *F_t_* decreases by 3.2%. This is because the laser energy is relatively low at this point, and as the input energy is insufficient, only a weak phase transformation is induced on the silicon carbide surface. The overall hardness and brittleness do not significantly reduce, with the material retaining its original high hardness and brittle structure. During grinding, the resistance to abrasive grain penetration and energy consumption for crack propagation is not significantly reduced, resulting in a small decrease in the grinding force.

When the laser current reaches 24 A, both *F_n_* and *F_t_* drop to the minimum value. Compared to the experimental group without laser modification, the *F_n_* is reduced by 17.6% and the *F_t_* is reduced by 22.2%. The trend of the actual values is consistent with theoretical values, but the theoretical values are larger. The maximum deviation is 32.1%, the minimum deviation is 5.8%, and the average deviation is 18.6%.

In summary, laser-modified silicon carbide ceramic grinding results in a significant reduction in both normal and Fts. With the increase in laser current, the laser power increases, the surface hardness of silicon carbide ceramics continuously decreases, and the corresponding reduction ratio of the grinding force gradually increases. The maximum reduction in *F_n_* reaches 17.6%, and the *F_t_* reaches 22.2%.

**Figure 9:**
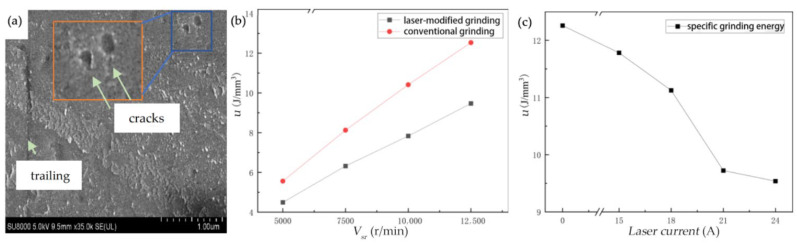
(**a**) SEM topography of the lapping surface. (**b**) The effect of *v_sr_* on *F_n_*. (**c**) The effect of laser power on *F_t_*.

The specific grinding energy *u* under *I*, *n*, *V_sr_*, and grinding depths *a_p_* was plotted as a dot–line graph. The curves showing a variation in grinding energy with laser power (indexed by current) and grinding wheel speed are presented in Figure 9.

## 4. Discussion

### 4.1. Thermal Distribution Simulation

The internal temperature of the laser-irradiated region in ceramic materials is related to the laser energy density $I_{abs}=A_{\lambda }\left(I\right)I_{\lambda }$, laser moving speed *v_p_*, laser spot diameter 2*r_b_*, laser irradiation time *t_p_*, and the material properties of the ceramic itself. Therefore, the material temperature is expressed by Equation (1):

(1) $$T=(I_{abs},t_{p},r_{b},v_{p},K,C)$$

Here, *K* denotes the thermal conductivity of the material, and *C* represents the specific heat capacity of the material. The spot diameter of the laser beam varies depending on the laser head and laser transmission method.

When the laser beam acts on the ceramic material, the laser absorptivity directly affects the effectiveness of material modification. Here, absorptivity *A* is used to represent the material’s absorption effect on the laser beam of a specific wavelength. The ratio of the laser energy effectively absorbed by the material *E_abs_* compared to the total energy emitted by the laser *E* is defined as the absorptivity *A*, as shown in Equation (2):

(2) $$A=\frac{E_{abs}}{E}$$

The total energy emitted by the laser beam equals the sum of the energy absorbed by the material, the energy transmitted through the material, and the energy reflected by the material. As SiC ceramics are translucent materials, the transmitted energy is assumed to be 0. Thus, the total energy E can be expressed by Equation (3):

(3) $$E=E_{abs}+E_{r}$$

*E_abs_* denotes the energy effectively absorbed by the silicon carbide ceramic; *E_r_* denotes the energy reflected by the silicon carbide ceramic; and *E* denotes the total energy emitted by the laser beam. Dividing both sides of Equation (3) by *E* yields Equation (4):

(4) $$1=\frac{E_{abs}}{E}+\frac{E_{r}}{E}=A+R$$

where *A* denotes the laser absorptivity and *R* denotes the laser reflectivity.

As reported in the literature [39], under normal temperature and standard atmospheric pressure, the laser reflectivity R of silicon carbide ceramics can be expressed by Equation (5):

(5) $$R=((n\;-\;\mu {)}^{2}\;+\;k^{2})/((n\;+\;\mu {)}^{2}\;+\;k^{2})$$

where *n* denotes the refractive index; *k* denotes the extinction coefficient; and *μ* denotes the magnetic permeability of the silicon carbide ceramic.

In conjunction with Equation (4), eliminating *R* yields the expression for laser absorptivity *A*, as shown in Equation (6):

(6) $$A=1-R=1-\frac{{\left(n-\mu \right)}^{2}+\kappa^{2}}{{\left(n+\mu \right)}^{2}+\kappa^{2}}$$

A pulsed laser rather than a continuous laser is adopted, so the laser energy appears periodically. In the nth pulse cycle, the laser power density function of the pulsed laser at the focal point follows Equation (7) [35].

(7) $$Q\left(x,y,t\right)=\left\{\begin{array}{cc} \frac{2\lambda P}{\pi r^{2}}\exp\left[-2\frac{{\left(x-x_{focus}\right)}^{2}+{\left(y-y_{focus}\right)}^{2}}{r^{2}}\right], & \left(n-1\right)\frac{1}{f_{laser}}\leq t\leq \left(n-1\right)\frac{1}{f_{laser}}+\tau \frac{1}{f_{laser}}+\tau \\ \;\;\;\;\;\;0, & \;\left(n-1\right)\frac{1}{f_{laser}}+\tau \leq t\leq \;n\frac{1}{f_{laser}} \end{array}\right.$$

where *Q* denotes the laser power density at spatial coordinates (*x*, *y*) and time *t* (typically expressed in units of W/m^2^). λ denotes the absorption coefficient of the laser. *P* is of the total pulse power of the laser (the output power of a single pulse). *r* is of the characteristic radius of the laser spot. (*x*_0_, *y*_0_) denotes the center coordinates of the laser spot (the position of the center point where the laser acts on the surface of the target material). *t* denotes time (the timing sequence of laser pulses). *n* denotes pulse number (the nth laser pulse). *f_laser_* denotes the pulsed laser frequency and *τ* denotes the pulse width.

**Figure 10:**
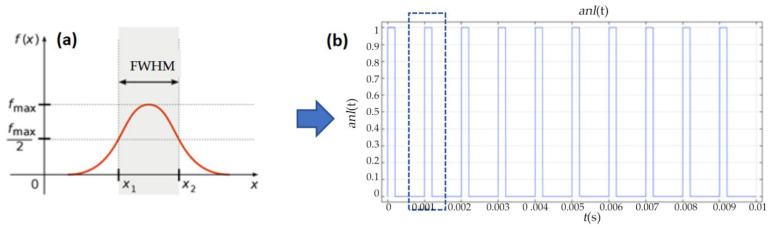
Characteristics of the laser source. (**a**) The time span of a Gaussian pulse. (**b**) The waveform of the square wave function.

The optical pulse duration is generally defined as the Full Width at Half Maximum (FWHM) of the laser power–time curve, i.e., the time span of a Gaussian pulse when its amplitude reaches half of the peak value, as shown in Figure 10a. A square wave function is used to replace the pulse width in the pulsed laser. To simulate the heat source of the pulsed laser, a periodic square wave function is configured, which satisfies the condition that the value is 1 during the pulse width time and 0 for the rest of the time within one cycle. The laser frequency is set to 1 kHz, one pulse cycle is 1 ms, and the pulse width is 0.2 ms. The waveform of the square wave function is illustrated in Figure 10b.

The temperature change in the material during laser irradiation is calculated according to the general heat transfer in Equation (8) [39]:

(8) $$\rho C_{p}\frac{\partial T}{\partial t}+\rho C_{p}u\cdot \nabla T+\nabla \cdot \left(-k\nabla T\right)=Q$$

where *ρ* denotes the density of the material SiC, $kg/m^{3}$. *C_p_* denotes constant-pressure specific heat capacity of SiC ceramic, $J\cdot {\left(kg\cdot K\right)}^{-1}$. *T* is of the transient temperature field of the material, K. *t* is the time variable, s. *u* is the velocity vector of the laser scanning system, $m\cdot s^{-1}$. The direction of *u* is consistent with the movement direction of the laser heat source relative to the workpiece. ∇ is of Hamiltonian operator. *K* is of the thermal conductivity of SiC ceramic, $W\cdot {\left(m\cdot K\right)}^{-1}$. *Q* denotes volumetric heat source term induced by laser irradiation, $W\cdot m^{-3}$.

The material parameters and laser parameters used for simulation are listed in Table 4.

**Figure 11:**
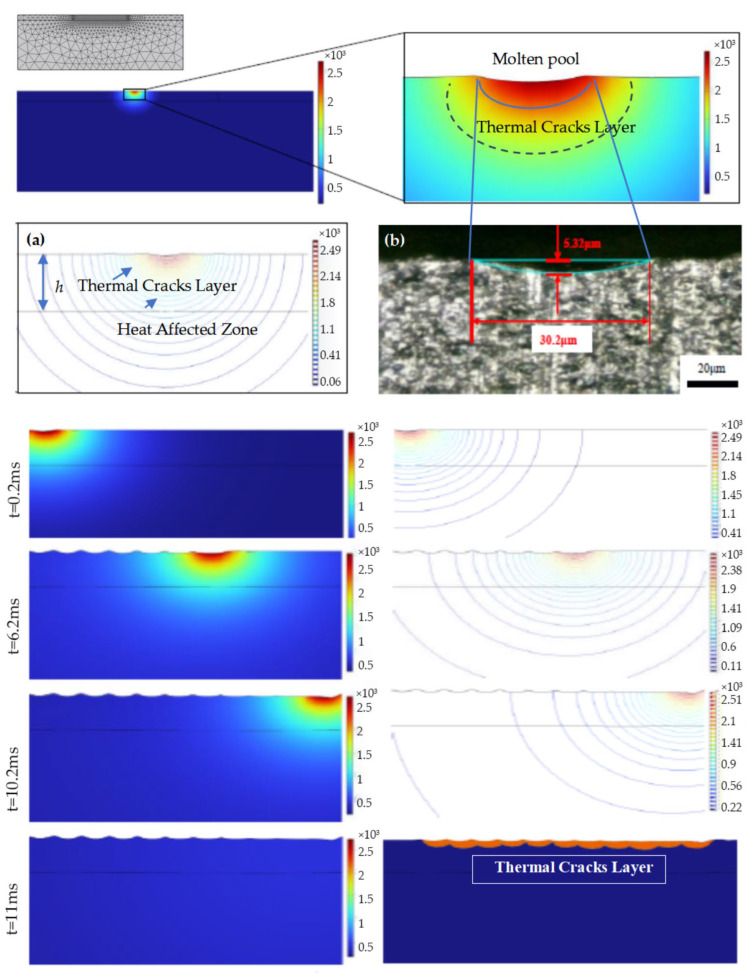
Simulation of laser irradiation on the SiC surface indexed by time. (**a**) Thermal field distribution. (**b**) Sample correlation with (**a**).

For the temperature field distribution, the laser beam energy is absorbed by the material, which generates a high temperature of over 2000 °C when the pulsed laser irradiates the silicon carbide surface. Based on the mesh shown in Figure 11, material ablation occurs when the temperature exceeds 2700 °C [35] and the corresponding mesh depresses. Since the energy of the pulsed laser beam follows a Gaussian distribution, a conical ablation crater is assumed to form on the surface. From the isotherm distribution diagram, it is clear that the high-temperature region generated by the material absorbing the laser beam energy is mainly concentrated in a semicircular area with a radius of 50 μm, centered at the laser focal point, while the temperature of other regions is less affected by the laser heat source.

A comparison between the cylindrical ablation crater generated in the simulation results and the crater measured in the laser irradiation experiment is shown in Figure 11. The images reveal that both have the same morphological characteristics, which is an arc shape with a deeper center and shallower sides. This corresponds to the energy distribution pattern of the Gaussian heat source.

### 4.2. Dimensions of the TCL

Crack Initiation Criterion: The balance needs to be upheld between thermal stress and fracture toughness. Experimental results show that the radial crack scale is much larger than the laser melting scale. When analyzing the crack region, material phase changes are neglected. For ease of calculation, the thermal expansion coefficient is assumed to be constant. Brittle materials have poor thermal conductivity, and the temperature gradient generated by laser heating causes uneven thermal expansion, which, in turn, induces thermal stress. Based on the theory of thermoelasticity, the model consists of two parts: stress calculation and the crack initiation criterion. The material is assumed to be linearly elastic and isotropic, and thermal stress is generated by “free expansion caused by temperature change and material constraint.” For a semi-infinite material heated by a circular laser spot, the radial stress σ_r_, hoop stress *σ_θ_*, and axial stress *σ_z_* are the main components.

Among them, the hoop stress is the key factor inducing radial cracks, and its distribution on the surface (*z* = 0) is expressed as follows:

(9) $$\sigma_{r}\left(r_{0}^{\prime }\right)\;=\;\frac{E\beta }{1-2\nu }\left[\int_{0}^{\infty }\left(1-\frac{r^{2}}{r^{2}+s^{2}}\right)T\left(s_{0}^{\prime }\right)sds-T\left(r_{0}^{\prime }\right)\right]$$

where *σ_r_* denotes radial thermal stress, ${MP}_{a}$. $r_{0}$ denotes characteristic radial position, μm. *E* denotes elastic modulus of the material, ${GP}_{a}$. β denotes linear thermal expansion coefficient, K^−1^. *ν* denotes Poisson’s ratio. *r* denotes integral variable (radial coordinate), μm. *s* denotes auxiliary integral variable (radial coordinate), μm. $T(s_{0}^{\prime })$ denotes instantaneous temperature at radial position *s*, K. $T(r_{0}^{\prime })$ denotes instantaneous temperature at the target radial position $r_{0}$. *s* denotes the integral variable corresponding to the radial position. When *r* < r_0_ (inside the laser spot), the thermal expansion of the material is constrained by the surrounding area, generating compressive stress. When *r* > $r_{0}$ (outside the laser spot), the unexpanded material is constrained by the internal region, resulting in tensile stress, the concentrated area of which is the region of crack initiation.

When the stress intensity factor *K_I_* induced by thermal stress at material defects (such as microcracks and impurities) reaches the fracture toughness *K_Ic_* of the material, cracks initiate and propagate. The core criterion is expressed as follows:

(10) $$K_{I\;}\geq {\;K}_{Ic}$$

**Figure 12:**
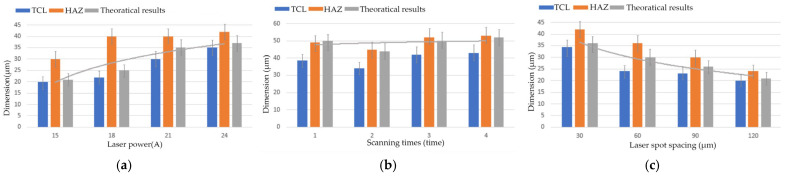
Dimensions of TCL, HAZ, and theoretically calculated value. (**a**) Plot of laser power vs. dimensions. (**b**) Plot of scanning times vs. dimensions. (**c**) Plot of laser spot spacing vs. dimensions.

For surface semi-elliptical cracks (a common crack topography induced by laser heating), the calculation formula for the stress intensity factor is expressed as follows:

(11) $$K_{I}\;=\;1.12\sigma \sqrt{\pi a}$$

where σ is the maximum tensile stress at the crack tip; *a* is half the crack length; *a* = 1 × 10^−6^ m; the shape factor of the semi-elliptical crack is 1.12; and $K_{Ic}$ = 4.5 MPa·m^1/2^. Calculated results are compared with experimental results, as shown in Figure 12.

## 5. Conclusions

Laser irradiation significantly alters SiC properties, affecting surface and subsurface cracks, hardness, phase composition, and topography.

The order in which the laser process parameters influence the dimensions of the surface cracks in SiC ceramics is as follows: laser power (indexed by current) > scanning times > defocus value > spot spacing. Additionally, the surface hardness of laser-modified SiC increases with the increase in the laser current and number of scanning passes and decreases at a higher defocus value and spot spacing, with a maximum reduction of 8.2%.

A large amount of SiO_2_ is formed in an oxide layer on the SiC surface after laser irradiation, with clear ablation groove boundaries. The width of the oxide layer increases with the increase in laser power and scanning passes and decreases at a higher defocus value and spot spacing. The SiO_2_ formed by oxidation has a lower hardness but higher fracture toughness than the SiC phase, which is conducive to improving the grinding conditions of SiC.

Laser irradiation results in the formation of a melting zone, a TCL, and an HAZ. The thicknesses of both the TCL and HAZ increase with the laser power and scanning times and decrease at a higher defocus value and spot spacing. Cracks are concentrated in the TCL. The maximum depth of the oxide layer reaches 20.13 μm, while those of the TCL and the HAZ reaches 41.21 and 53.84 μm, respectively.

## Figures and Tables

**Table 1:** Performance parameters of silicon carbide ceramic materials.

Parameter	Value
Density (kg·cm^−3^)	3.12
Elastic Modulus (GPa)	415
Vickers Hardness (HV)	3000
Fracture Toughness (MPa·m^1/2^)	4.5
Thermal Conductivity (W/m·k)	148
Melting Point (°C)	2700
Poisson’s Ratio	0.24
Thermal Expansion Coefficient (×10^−6^ °C^−1^)	4.2

**Table 2:** Main technical parameters of laser engraving machine.

Parameter	Value
Wavelength *λ* (nm)	532
Beam diameter *D* (μm)	50
Maximum scanning speed *V* (points/s)	1000
Maximum output power *P* (W)	5
Frequency *f* (kHz)	1
Single-pulse energy *Q* (mJ)	2.5
Maximum scanning area *S* (mm^2^)	90 × 90 × 90

**Table 3:** Main grinding parameters of laser-modified SiC.

Parameter	Value
Wheel speed *V_sr_*, r/min	5000, 7500, 10,000, 15,000
Worktable speed *V_w_*, mm/min	2.5, 5, 7, 10
Grinding wheel	Electroplated diamond
Grain size, μm	38

**Table 4:** Material and laser parameters.

Parameter	Value
Spot Radius (μm)	45
Scanning Speed (mm/s)	60
Melting Point (°C)	2700
Heat of Sublimation (kJ/kg)	11,562.5
Density (kg/m^3^)	3200
Laser Frequency (kHz)	1
Pulse Width (s)	0.0002
Surface Emissivity	0.79
Thermal Conductivity (W/(m·K))	236
Constant-Pressure Specific Heat Capacity (J/(kg·K))	680
Ambient Temperature (°C)	20

## Data Availability

The original contributions presented in the study are included in the article, further inquiries can be directed to the corresponding author.
